# Assessing the costs of historical inaction on climate change

**DOI:** 10.1038/s41598-020-66275-4

**Published:** 2020-06-08

**Authors:** Benjamin M. Sanderson, Brian C. O’Neill

**Affiliations:** 1 CERFACS, Toulouse, France; 2 National Center for Atmospheric Research, Boulder, CO USA; 3 University of Denver, Denver, CO USA

**Keywords:** Climate-change mitigation, Projection and prediction

## Abstract

We consider alternative history scenarios in which explicit climate mitigation begins before the present day, estimating the total costs to date of delayed action. Considering a 2(1.5) degree Celsius stabilization target, peak costs are greater and reached sooner with a later start to mitigation, reaching 15(17)% of global GDP in 2085(2070) for a 1990 start and 18(35)% in 2080(2035) for a 2020 start. Further mitigation delay costs a best estimate of an additional 0.5(5) trillion dollars per year. Additional simulations show how optimal mitigation pathways evolve without imposing a warming limit, finding that median abatement levels and costs are not strongly dependent on start date. However, whereas 18(5) percent of optimal solutions starting in 1980 meet the 2(or 1.5) degree target, 5(or 0)% of 2020 simulations meet the goals. Discounted damages due to delayed mitigation action rise by 0.6 trillion US dollars per year in 2020.

## Introduction

In order to make informed decisions on future emissions and climate adaptation policy, it is necessary to establish a framework which is capable of estimating the costs and benefits of different possible future actions. Such a framework is required to justify actions or in-actions in the present informed by potential costs of mitigation, adaptation, or impacts which might be incurred in the future. Such a process also allows the potential costs arising from greenhouse gas emissions to be incorporated into an established economic system, by defining a “social cost of carbon” quantifying future damages (costs of impacts) from present day emissions^1,2^.

**Figure 1:**
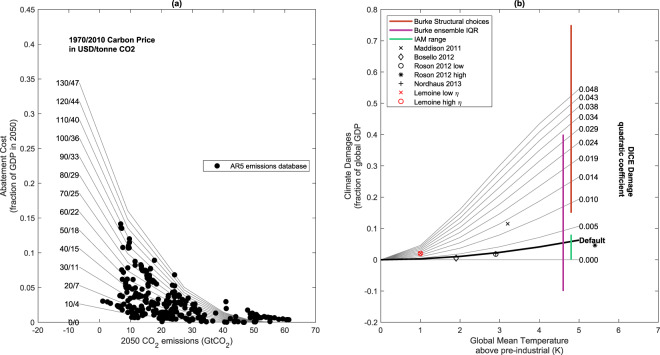
Illustration of calibration for the (**a**) abatement backstop and (**b**) climate damage quadratic coefficient parameter range in the parameter ensemble. Points in (**a**) show each available member of the AR5 parameter ensemble which reports both emissions and costs as fraction of GWP in 2050. Curves show a parameter sweep of the initial 1970 backstop cost using the range in Table 1. Backstop costs decline exponentially by year at the default DICE rate of 2.5% per year - such that 2010 values are 36% of the 1970 values. Black lines in (**b**) illustrate the climate damages (expressed as a fraction of GWP) associated with different levels of global mean warming above pre-industrial, using the default DICE quadratic damage function. The quadratic coefficient is modulated according to the range in Table 1 to produce the family of curves illustrated here. The range is adjusted empirically to be representative of uncertainty in climate damages at 5C of warming relative to pre-industrial using estimates from^5^. Central estimates of GDP impacts at different warming levels are shown from a number of studies for context^10–15^.

A subset of integrated assessment models (IAMs), so-called benefit-cost IAMs^3^, are the primary type of research tool used to evaluate multiple costs and benefits of climate change policies^4^. Their representation of economic damages from climate change impacts, however, is usually defined as aggregate relationships between global average warming levels and damages whose parameters are defined by meta-analysis^5^. These “damage functions” are uncertain: there is physical model disagreement on how some key future economic damages such as extreme rainfall will scale with temperature^6^, and Earth System Models generally lack the complexity to fully resolve the dynamic ice-sheet processes necessary to capture the range of possible sea level rise^7^. From a socio-economic perspective, there is wide uncertainty in how changes in the climate system translate to impacts on the economy, and how that translation will depend on the changing vulnerability of society over time^8,9^. Damage functions can be estimated either on the basis of “bottom up” sectoral impact projections^10–14^ or by “top-down” methods based on statistical analysis of historical temperature-GDP relationships^15,16^. Representing damages at the process level is done in some “detailed process” IAMs for specific sectors (Weyant, 2017). Fuller incorporation would require coupled consideration of human and physical systems which influence factors such as agricultural productivity and habitability of environments^17,18^ and such capacity, where it exists at all, only describes a subset of human-climate interactions^19^. As a result, a wide range of climate damage functions can be justified in economic models^20^, and damages for a given level of warming can vary by an order of magnitude depending on the assumptions made^21^. The damage function in the DICE (Dynamic Integrated Climate-Economy) 2013R model used in this study^22^ can be modulated to span the range of damages observed in the literature (see Fig. 1(b)).

IAMs also need to represent mitigation of greenhouse gases in the context of a set of model variables. This implementation varies by IAM, subject to assumed technological^23^ and economic pathways^24^ and baseline characteristics^25^ - which can result in a wide range of possible costs for achieving a given abatement level. In DICE 2013R, mitigation costs are represented in an aggregate sense by a set of exogenous and endogenous variables. The former include a marginal abatement cost function describing the cost of each additional unit reduction of emissions relative to a baseline case in which no mitigation occurs, including the cost of a backstop technology, defined as the cost of a generic zero-carbon technology assumed to be available in the future in unlimited supply if abatement costs become high enough. The level of abatement, including use of the backstop technology, as a function of time is then determined in the process of optimizing the scenario pathway. By sampling the marginal abatement cost function parameters in DICE, a range of costs can be simulated consistent with the uncertainties in the IAM literature (see Fig. 1(a)).

Optimization objectives can be either to minimize combined discounted damage and mitigation costs, or to minimize costs while achieving a desired target climate pathway (such as meeting a temperature target)^20^. It has been noted that this framework is an idealization; the concept of a baseline future without climate policy or impacts becomes less meaningful as mitigation technology is implemented and society experiences costs associated with climate change^26^.

A wider conceptual question also exists whether existing economic principles are appropriate for climate change. A traditional exponential discount rate might be appropriate for representing present and future costs for a single person^27^, but for the climate problem, century scale changes raise questions of inter-generational fairness^17,28^ which some have attempted to resolve through an ‘inter-generational discounting’ rate which explicitly resolves benefits and costs for those not yet born^29,30^.

Most IAM applications simulate current and future policies which represent a possible joint evolution of climate and society^24^. For example, the major IAMs used to produce emissions and land use scenarios to drive global climate models in big international coordinated efforts, as for producing CMIP (Coupled Model Inter-comparison Project) ensembles, which in turn inform the IPCC (Intergovernmental Panel on Climate Change) process, are generally initialized in a present-day, or near present-day, state.

There are some questions, however, for which a retrospective approach might be useful. Hindcasts, by which we mean model projections that begin in a historical year and are then compared to actual experience since then, can be used to evaluate model performance, to improve understanding of the effectiveness of alternative policy options including those related to the timing of actions^31^, and to inform issues related to accountability and responsibility for damages. For example, hindcasts of how stratospheric ozone concentrations (and their climate implications) might have evolved with alternative CFC policies have improved understanding of policy approaches to that issue^32^. Similarly^33^, investigated the relevance of the timing of the start of negative emissions as an approach to returning CO_2_ to pre-industrial levels by simulating the effect of a delay in such measures from 2013 to 2021.

Regarding the historical responsibility for damages, the ethical framework for such issues is a subject of research and debate^34^, requiring the joint consideration of past and future emissions, compensation for potentially uncertain impacts and an assessment of what is fair. Although a discussion of the ethical considerations is not the subject of this paper, establishing responsibility for damages which have arisen as a result of the action or inaction of an individual, nation state or company will require (amongst other things) a practical assessment of what impacts might have occurred in a counter-factual world where other choices had been made^35^.

In the following paper, we attempt a more general discussion of this concept by developing a set of alternative histories where significant global climate mitigation had begun at various points in the past. Using this approach, we seek to address a number of key questions: first, within a cost-effectiveness framework, how might the costs of mitigation to reach the temperature goals of the Paris Climate Accord have differed, had a least-cost emissions pathway been enacted beginning at alternative points in the past, and what is the ongoing annual cost of delay in adopting a least-cost decarbonization pathway. Secondly, within a cost-benefit framework without a pre-defined temperature target, but with a joint assessment of mitigation costs and climate damages, how would the optimal pathway have evolved given an initialization of climate policy in a past year.

This study uses DICE 2013R as implemented in MATLAB by^20^. The model structure, parameter choices, and implementation of cost-effectiveness and cost-benefit simulations are described in section 4. Briefly, the model is simple and globally aggregated, but nonetheless represents in an integrated fashion the key elements necessary to illustrate the type of analysis that can be brought to bear on these questions, including a representation of the drivers of future emissions growth, the climate response to emissions, mitigation costs and options, the costs of impact-related damages to the economy, and discounting and intergenerational issues.

## Analysis

Clearly, each element of this model is subject to large parametric and structural uncertainty^36–38^. We partially address the former by perturbing key parameters controlling the social rate of time preference, climate sensitivity, mitigation costs^38,39^ and, in the latter section of the paper, climate damage costs^20^ (see Table 1 for values and ranges used). Uncertainty in mitigation costs and climate damages are sampled by parameters which linearly modulate the DICE abatement backstop costs and default damage function (see Fig. 1 and Section 4).

**Table 1:** Parameter Ranges for the DICE model used in this study.

Parameter	Units	Default	PDF	Lower bound	Upper bound
Climate Sensitivity	K	2.9	^51^	—	—
Climate Damage Quadratic Coefficient	f(GWP)K^−2^	0.218	flat	0	3.924
Cost of backstop decarbonization in 1970*α*	USD/tonne CO_2_	flat	n/a	0	130
Initial rate of Social time preference per year	—	0.015	flat	0	0.02

Lower and upper bounds indicate limits of the flat PDF used to represent parameter uncertainty, if appropriate.

The baseline scenario (in which there is no climate damage, and no mitigation undertaken) is based on that used for the 2010 US Department of Energy assessment of the social cost of carbon^40^ (described in Section 4.2 and illustrated in Supplementary material Fig. A.1), but each parameter configuration has its own effective baseline and costs are calculated for each parameter set relative to that baseline. The configuration of the model is discussed in Methods section 4.

All the simulations considered here start in the year 1970. Delayed mitigation simulations are implemented by fixing the DICE emissions control rate (MIU) at zero until a given date, after which the model can freely choose that control rate for each year of the simulation. Optimal solutions are found by maximizing the 1970 present value of welfare over the entire simulation period (that is, the discounted sum of welfare from 1970 through 2150, the end of the simulation period). Welfare is negatively impacted by both emissions reduction costs and by climate damages.

Although we address some of the model’s parameter uncertainty, we do not in this study address the structural assumptions or functional forms of the default DICE model or aspects of the baseline scenario such as population growth, labor productivity, or rates of technological change. We recognize that any comprehensive assessment of costs would need to consider a super-set of available, plausible economic and physical representations of the system.

**Figure 2:**
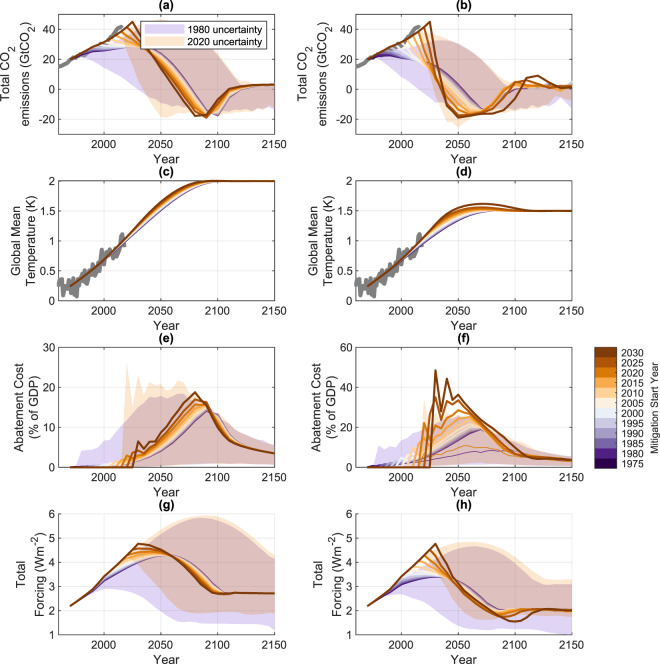
Evolution of least cost emission pathways for counterfactual histories meeting the Paris temperature targets and departing from the baseline scenario at various times. Solid lines show the default DICE response, shaded areas indicate the 10th and 90th percentiles of the ensemble distribution, indicating parametric uncertainty in representative simulations beginning in 1980 and 2030. (**a**,**b**) show the total CO2 emissions evolution for a (2C,1.5C) target, (**c**,**d**) show the accompanying temperature evolution and (**e**,**f**) show the annual undiscounted total abatement costs (climate damages are not considered in this section). (**g**,**h**) show total climate forcing. Colored lines show evolution for different counterfactual historical start dates. Grey lines show observed historical values^55,56^.

The first set of calculations use the model to find (if possible) a solution meeting (and not exceeding) each of the Paris global average temperature goals of 1.5 and 2.0 °*C* (see Methods section 4.3). Climate damage costs are not considered in this section, given the model’s damage function is zero by construction at temperatures below the target. The results are presented in Fig. 2. For both the 1.5C and 2.0C targets, the least-cost pathways show an evolving tendency such that a later start date requires both more rapid near-term emissions reduction (decarbonization) and a commitment to larger (for the 1.5 degree target) and earlier negative emissions later in the century.

**Figure 3:**
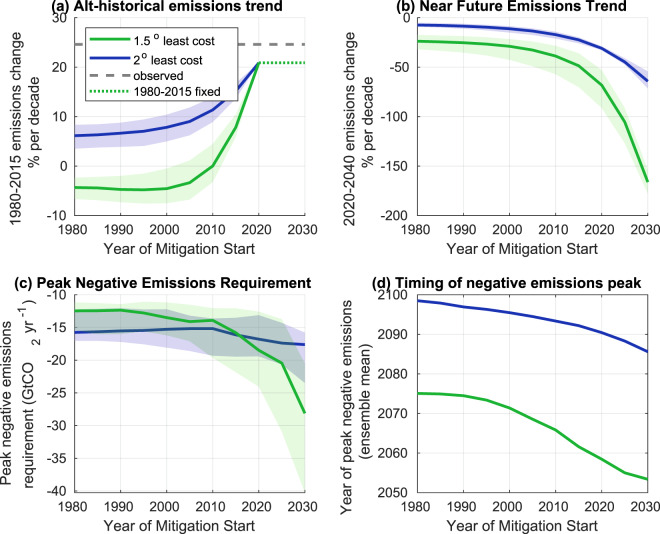
An illustration of (**a**) 1980–2020 and (**b**) 2020–2040 emissions trends required for least-cost 1.5 and 2.0 degree non-exceedance cases as a function of abatement start date. The peak rate (**c**) and time (**d**) of negative emissions are also shown. Solid lines show the ensemble median (**a**–**c**), (d is a mean over discrete values), while shaded regions show (**a**–**c**) show the 10th and 90th percentiles of the perturbed ensemble distribution.

The default model simulation for the least-cost 2C pathway starting mitigation in 1980 would have allowed an average increase of global average CO_2_ emissions of 6% per decade from 1980–2020, compared with the real world average increase of 24% per decade (Fig. 3(a)). For 1.5 degrees, a least-cost scenario with abatement starting in 1980 would have exhibited a 5% per decade reduction from 1980 to 2015. Later mitigation start times imply a greater near-term commitment to decarbonization (Fig. 3(b)). A least-cost pathway for 2(1.5) degrees starting in 2020 requires default 2020–2040 emissions reductions of 30(70)% per decade. A further 5 year delay increases the least-cost 2020–2040 decarbonization trends to 45(105%) per decade (implying a near instantaneous transition to negative emissions in the 1.5C case).

The model generally deploys negative emissions earlier as the mitigation start time advances, with peak rates of negative emissions for 2C in 2100 for a 1980 start date, and 2090 for a 2020 start date. The peak rate of removal does not change markedly in the range of start dates considered here for 2C. However, delaying mitigation in the least-cost 1.5 degree solutions both increases likely peak removal rates and hastens the time of deployment from 12 GtCO_2_/yr in 2075 for a 1980 abatement start to 18 GtCO_2_/yr in 2058 for a 2020 start (Fig. 3(c,d)).

Figure 3(b) implies that least-cost post-abatement decarbonization rate is not highly sensitive to the parameter values sampled (climate sensitivity, discount rate or cost of mitigation technology), although the long term negative emissions commitment is. This is broadly consistent with assessments which find that near term climate policy decisions are not highly dependent on climate sensitivity^41^, but also implies that least-cost pathways are not strongly conditional on economic parameters in the near term. The peak negative emissions requirements, however, are quite uncertain - varying from -12GtCO_2_/yr to -25GtCO_2_/yr for a 1.5C least-cost pathway beginning mitigation in 2020.

For each start date we also produce a range of possible costs for meeting the temperature targets, where annual costs are shown as a fraction of Gross World Product (GWP) in Fig. 2(f,g) for the 2C and 1.5C targets respectively. Simulations in which abatement starts later exhibit both a more rapid rise in costs, and greater mid-century peak. For example, for a 2020 abatement start for a 2C least-cost target, costs rise within 5 years to 3 percent of GWP (10–90 percentile range of 2–5% of GWP), ultimately peaking at a central estimate of 16% (4–21%) by 2070. In contrast, a least-cost scenario meeting the 2C target beginning in 1980 would have taken 50 years to reach 3% of GWP, with emissions peaking later at 13% of GWP in 2080 (with an 10–90 percentile uncertainty range of 2.5–16% of GWP) (Fig. 2(f)).

Costs for the 1.5C target are significantly greater. For a 2020 initialization, costs by 2025 reach 20% of GWP, with an 10–90 percentile uncertainty range of 15–30% of GWP), peaking in 2050 at 25% (8–35%) of GWP. By contrast, a least-cost 1980 abatement start would have had a median abatement path that never reached 20%, but rather slowly increased to a peak cost of 18% by 2080.

**Figure 4:**
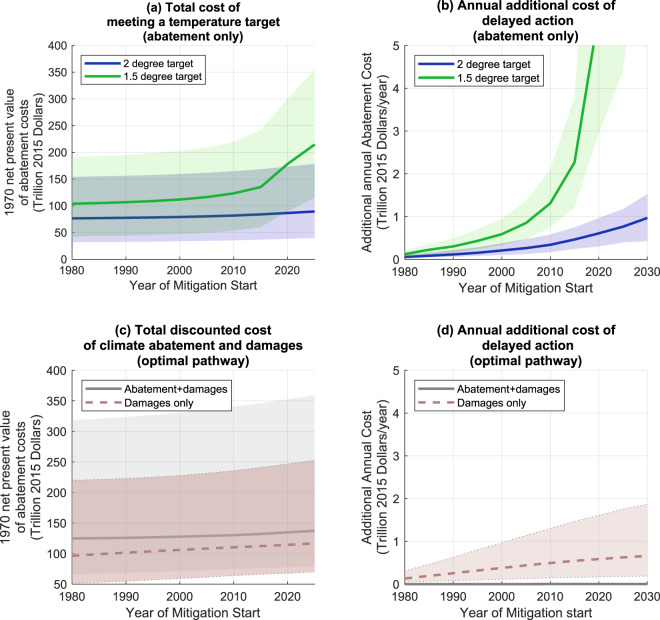
(**a**,**b**) Net present value of abatement costs as a function of the year in which abatement begins in temperature target experiments. Costs are calculated as the sum of the discounted difference in total GWP (from 1970 to 2150) between least-cost 1.5 or 2 degree scenarios, and a scenario with the same parameter values without climate mitigation or damage costs. (**a**) shows abatement costs in 2017 US Dollars for non-exceedance of 1.5 or 2 degrees C above pre-industrial conditions. Solid lines show the ensemble 50th percentile, while shaded areas show the range between the 10–90th percentiles. (**b**) shows the range of increase in costs associated with a day’s delay in mitigation. (**c**,**d**) as for (**a**,**b**) for optimal simulations where model is used to find the optimal trade-off between damages and abatement costs, while abatement spending is constrained to zero until the mitigation start date. Solid grey line shows the ensemble 50th percentile, while dashed red line shows the portion attributable to climate damages alone. Shaded areas show the 10th and 90th percentiles of the ensemble distribution.

Figure 4(a) shows the evolving increase in the net present value of discounted abatement costs for the least-cost scenarios which meet 1.5 or 2.0C targets. The total discounted costs for the 2C least-cost pathway increase as mitigation is delayed (Fig. 4), but not dramatically. Median discounted total costs for a 2C target increased by about 20% between 1980 and 2020. This increase reflects two competing effects: delaying mitigation decreases short-term costs by definition, because it makes them zero before mitigation begins, while it increases costs in the longer-term (Fig. 2(e)) because higher rates of emissions reductions are required due to the later start. Costs for a 1.5 degree target grow much faster; the delay from 1980 to 2020 increases abatement costs by about 100%, with 40% increase between 2010 and 2020 alone, reflecting a change at that time to a sharper increase in the cost of emissions reductions needed to make up for the later start.

There are large parameter uncertainties associated with these results, but the sign of the cost of delay is unambiguous: for a 1980 start, total discounted post-1980 costs for meeting 2C would range from 40 to 150 trillion 2015 dollars (10th and 90th percentiles). The median rates of change of costs in meeting the targets are rising: costs for meeting the 2C target rose by 0.3 trillion dollars per year in 2010, with the annual cost increase rising to nearly 0.6 trillion dollars per year in 2020. The cost of meeting 1.5 degrees rises dramatically - from 1.3 trillion dollars per year of inaction in 2010 to over 5 trillion dollars per year in 2020. These numbers are broadly comparable with the findings of^31^, who estimated a cost of order 0.5 trillion dollars per year for a 2 degree target (measured from 2005).

### Optimal simulations with climate damages

A second set of simulations uses DICE in its unconstrained configuration without a temperature target imposed, using a quadratic damage function which smoothly increases as a function of global mean temperature, representing economic damages which would be experienced due to climate change. We discuss the methodology of these simulations in section 4.4, where we sample a wide range of possible coefficients for the default DICE damage function.

**Figure 5:**
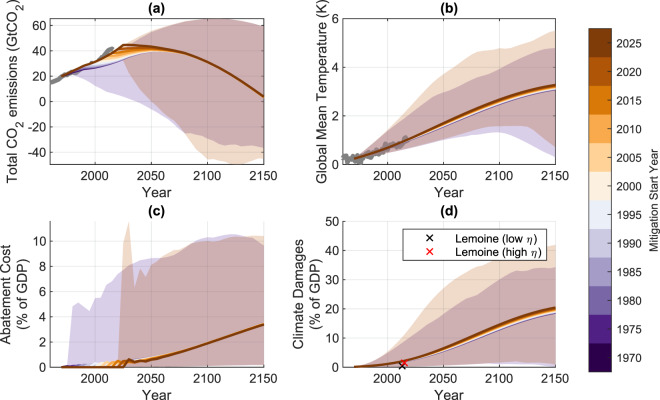
Evolution of optimal emission pathways for counterfactual histories with a range of mitigation start dates. Solid lines show the default DICE response, shaded areas indicate the 10th and 90th percentiles of the ensemble distribution, indicating parametric uncertainty in representative simulations beginning in 1980 and 2030. (**a**) shows the total CO2 emissions evolution, (**b**) shows the accompanying temperature evolution, (**c**) show the annual total abatement cost and (**d**) shows annual climate damage costs. Colored lines show evolution for different counterfactual historical start dates (future dates departing from the baseline are shown as dashed, where the model is able to solve). Grey lines show observed historical values for CO2 concentrations^56^ and temperature^55^. Red and black crosses show historical estimates of climate-impact costs at 1 degree warming^15^ for low (*η* = 1) and high (*η* = 4) tolerance of global inequality.

The results are shown in Fig. 5. Earlier times of adoption of optimal pathways have a greater effect on the lower uncertainty bound than the upper bound of the distribution. If adopted in 1980, the lower bound of warming limits temperatures to 1.3C above pre-industrial conditions. However, the 10th percentile of maximum warming in the ensemble in the simulations initiated in 2015 is 1.9C.

An optimal solution in which action was taken beginning in 1980 would have involved reducing the rate of growth in global emissions until a peak was reached around mid-century. With mitigation starting in 2020, emission growth essentially stops now; emissions immediately stabilize and remain constant before declining beyond mid-century. The delay in mitigation from 1980 to 2020 implies optimal future pathways with somewhat higher global temperature and climate, translating into an increase in likely 2100 damages of about 15%.

However, without strict temperature constraints, the median and upper bounds of the distributions are not strongly affected by the delayed start in adoption of the least cost pathway. The default model version does not show a large dependency of the temperature trajectory as a function of the time at which abatement is enabled, increasing by only a small amount with a later adoption of the optimal pathway (Fig. 5).

The evolving discounted costs of the optimal solution are shown in Fig. 4(c,d). Unlike the constrained least-cost case, there is no rapid increase in median abatement costs with delayed mitigation. This is largely due to the fact in most parameter configurations, there is only limited pre-2020 abatement spending in the optimal solutions (Fig. 5(c)). However, there is a increase in climate damage costs as the time of mitigation starting moves forwards, with a median increase in discounted future damages of 0.6 trillion USD per year in 2020 for each year of delayed mitigation (with 10th/90th percentiles of 0.1/1.6 trillion USD per year).

**Figure 6:**
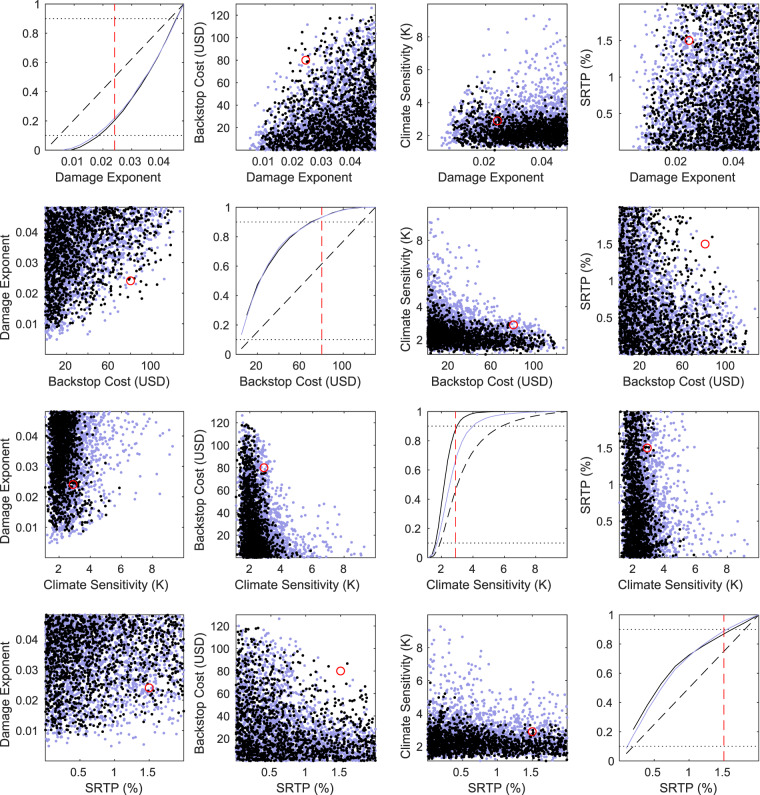
A multivariate distribution of ensemble members which do not exceed 2 degrees of warming, and initiate a least cost pathway in 1980 (blue) and 2020 (black). Each off-diagonal scatter plot is a function of two input parameters in the perturbed ensemble. Only simulations which do not exceed 2 degrees are shown, thus illustrating the regions of parameter space which would support the target being met. On diagonal plots are normalized cumulative histograms of the value of each of the four input parameters. Solid lines are the cumulative histograms of the simulations which do not exceed 2 degrees, while dashed histograms are the prior distributions of all ensemble members (including those which exceed 2 degrees warming). The red circle shows the default model parameters used for the main lines in Figs. 2 and 5.

We can explore the conditions necessary for a 2 degree target to be met from 1980 and 2020 conditions in the optimal, unconstrained solutions with quadratic damage functions by considering the regions of the ensemble parameter space which support a sub 2 degree maximum warming (before 2150). Figure 6 illustrates this space, showing that only certain combinations of parameters allow the target to be met without a hard constraint.

Climate sensitivity (*S*) exerts a strong control on the feasibility of the target. The 90th percentile of the prior ensemble distribution on *S* is 5.6 K, but 90 percent of simulations meeting the 2 degree target for abatement starting in 1980 have *S* < 4 K, and 2.9 K for an abatement start in 2020. Simulations with a larger damage exponent, lower abatement costs and lower social rate of time preference are more likely to meet the target.

## Discussion

We have considered here the question of the value of lost time. How much more efficiently could humanity have addressed climate change if a different pathway had been taken in the past? We acknowledge that a comprehensive answer to this question may be impossible to attain - a counterfactual history could have differed in countless ways, with many complex socioeconomic or political feedbacks which could never be captured in a model as simple as the one used in this study. Some of the findings may prove useful - which we discuss before considering caveats.

First, seen through the lens only of the Paris temperature goals, the lost time since the 1980s has doubtless made it more difficult (or perhaps impossible, in the case of the 1.5C goal) to meet them. A least cost 2C scenario initiated in the 1980s would have involved several decades of gradual decarbonization, followed by a commitment to net zero emissions late in the 21st century. In contrast, a scenario starting from present day conditions requires a halving of global emissions in little more than 15 years, and net zero emissions by 2060. Mitigation costs for achieving the 2 degree target have risen as a result of the delay. While the expected total (discounted) costs over time have increased modestly (10% or so), the later start necessitates very large near term costs (up to 3–5% of GWP by 2025). Such costs continue to climb rapidly with any further delay.

Uncertainties in the costs are high, but the annual increase in cost is relatively well constrained - with each additional year of delay in implementing mitigation costing an additional 0.3–0.9 trillion dollars in total discounted future mitigation costs, if the 2C target is to be ultimately met.

The simulations constrained to meet the 1.5C goal suggest that, with the assumptions and model used here, the goal requires near-term spending which may not be feasible: a 2020 start to abatement would require 15–30 percent of GWP by 2025. In contrast, had abatement started in 1980, costs could have risen gradually over more than 80 years and peaked at less than 20% of GDP.

Another perspective on the implications of delay is provided by simulations that are not constrained by temperature targets but that identify optimal pathways balancing mitigation costs with damages from climate change impacts. Optimal simulations with abatement starting in the present day show a much more rapid rate of change in emissions than simulations starting in 1980, and the optimal solution results in higher long term climate damages. In the optimal solutions, the majority of the costs of mitigation delay come in the form of additional climate damages.

The optimal solution only meets the Paris targets in simulations with low decarbonization costs, low climate sensitivity, high climate damage coefficient and with a small discount rate. The majority of optimal solutions which do not exceed the 2 degree threshold have climate sensitivities of 2.9C or less, implying that the upper end of the IPCC’s 1.5 to 4.5C uncertainty range^42^ is incompatible with the 2 degree target. 90 percent of solutions require mitigation costs low enough to be consistent with a backstop price of 70 USD/tonne, significantly less than the value assumed in the US SCC calculation^40^ of 344 USD/tonne of CO2. Mitigation costs of this magnitude are not, however, out of the range of published IAMs, a selection of which achieve comparably low mitigation costs (see Supplementary Material, Table A.1).

The lower bound of warming is increased in the simulations adopting the unconstrained optimal pathway in 2020 rather than 1980: 18 percent of the ensemble starting mitigation in 1980 have optimal solutions with less than 2 degrees of peak warming, compared with 5 percent for a 2020 mitigation start. However, the effect on the mean and upper bound of the distributions is muted.

In most parameter configurations, mitigation costs are pushed to the future when backstop prices are lower. This occurs partly due to discounting, which decreases the weight given to future costs. In addition, given the model’s assumption of perfect foresight, when it is known that mitigation costs in the future will decline, the optimal solution delays mitigation to take advantage of that knowledge. The perfect foresight assumption may also affect optimal near term emission policy by not accounting for the possibility that future learning may lead to damages or mitigation costs that turn out to be unexpectedly high. Existing work on the effect of learning has not generally demonstrated a large effect on optimal policies in the near term (e.g.^10^), partly because that learning may work in either direction, leading to unexpectedly low (and not only higher) costs. However, asymmetric effects of learning in either direction could change such conclusions.

There are also several uncertainties not explored here that could affect conclusions. First, the relation between CO2 emissions, concentrations, and temperature change is important to cost results and subject to structural uncertainty. With the DICE2013R carbon cycle and climate model, the stabilization scenarios used here require in some cases large net negative emissions flux at constant temperatures (see Fig. 1), whereas some former studies have found that near-zero net emissions are sufficient to stabilize temperatures^43^. Quantifying this ‘zero-emissions commitment’ is a subject of active study^44,45^ - and remains an uncertainty in our conclusions. To the extent that the negative emissions required for stabilization are over-stated, our mitigation costs would be over-estimated.

Second, costs are also affected by the assumption that CO2 and non-CO2 abatement are correlated. While this serves as a rough proxy for a muti-gas mitigation approach, other approaches assume limits to non-CO2 reductions^46^. To the extent that our assumed potential for non-CO2 reductions is too large, our mitigation costs would be under-estimated.

Finally, we acknowledge some sociological assumptions - firstly, that society would have known at past times that the Paris targets might be desirable. This is clearly debatable, from the societal standpoint, for 1980, when there was little consensus on global climate sensitivity or impacts. Recent analysis of publicly available documents, however, have shown that the potential gravity of human-caused climate change was known by many companies as early as that date^47^. In the policy debate arena, it could be argued that there was global consensus for the need for climate mitigation by the early 1990s^48^. We also do not explore sociopolitical uncertainty in the baseline scenario (i.e. the evolution of emissions, population and GDP in the absence of climate policy). As such, the uncertainties explored here (broadly climate response, mitigation and damage costs) are a subset of a potentially wider set of uncertainties relating to humanity’s ability to predict the physical and economic evolution of the planet.

Despite uncertainties associated with the absolute costs, the results of this study highlight some general aspects of the decision-making framework which are likely robust. The difficulty and cost associated with delayed action towards meeting a temperature target are likely to increase over time, and that rate of increase has the potential to be strongly nonlinear. In the framework considered here, the 1.5 degree temperature target is already in this regime and total costs of meeting the target are considerably greater (and less certain) than they would have been if stronger past mitigation action had occurred.

This paper, therefore, represents an attempt to apply a retrospective lens to global integrated assessment modeling. We acknowledge that the model and analysis are relatively simple, but we believe there is value in this endeavour; looking back can help us build models which better reflect reality, allow us to frame decisions which lie in the future, and to detangle how we consider responsibilities for past actions.

## Methods

We use a MATLAB implementation^20^ of the DICE 2013R global coupled climate and economy model^22^ (the MATLAB version used here was developed in 2012^20^, but the codebase^49^ has been updated to the 2013R parameters). The model has been used extensively since its introduction in the early 1990s, and recently in assessments of emissions pathways necessary to achieve the Paris temperature goals^50^.

The model solves iteratively for a pathway that maximizes inter-temporal social welfare (itself a function of consumption), given assumptions about population and labor productivity which drives economic growth, discounting, costs of emissions reductions and a function relating global mean temperature to climate damages on the economy. Optimal solutions are calculated by identifying pathways of savings rates and emissions reduction rates that maximize the discounted sum of social welfare over time. This solution produces an emissions pathway that effectively minimizes combined damages and mitigation costs. DICE has perfect foresight, so decisions about savings and emissions reductions are made in the full knowledge of how the price of mitigation will evolve in the future, and how costly different levels of climate change will be. This is clearly a simplification (although not an uncommon one) of much more complicated types of knowledge and uncertainty about the future, and we discuss its implications in the main text.

Baseline emissions are those that result from the optimal solution to the model when it is assumed that there are no climate damages and no emissions mitigation. The baseline scenario for our default model parameters follows^40^, and is illustrated in Supplemental Fig. A.1. Emissions abatement is calculated as a fraction of the baseline emissions, and is controlled by an emissions control rate (MIU^22^), which we fix at zero until the time at which mitigation begins. After the mitigation start date, MIU is allowed to increase such that emissions can be reduced compared with the baseline (allowing for negative emissions if MIU is greater than 1). DICE has an exogenous representation of carbon emissions per unit economic output, and the economy can pay for additional emissions reductions by means of a mitigation module in which the cost of emissions reduction is a function of the control rate. Costs are also influenced by the assumed price of a backstop clean technology which starts from an initial cost *α* and decays over time at a fixed rate (in this study) of 2.5% for each 5 year period^36^.

Land use emissions are exogenously defined in the model, non-CO_2_ forcing agents are represented in a bulk form, and their emissions are scaled by the same abatement factor as CO_2_. The climate response is simulated by a global energy balance model with a two-layer diffusive ocean as per the default configuration in^22^ (with the exception of the climate sensitivity parameter, which is explicitly perturbed). All model simulations begin in 1970 and are run through 2150.

### Parameter sampling

In each section of the study, we perform a 1000 member perturbed ensemble using a Latin hypercube sampling strategy over the parameter space defined in Table 1. A flat prior is used for social rate of time preference, backstop cost and damage coefficients, whereas an empirical PDF from^51^ is used for Equilibrium Climate Sensitivity. We use this ensemble to produce a diverse range of baseline and counterfactual simulations, conditional on the parametric PDFs used. Each parameter configuration is used for a set of simulations, all initialized on the same date (1970) - but differing in the date at which abatement is allowed to have a non-zero value, and in the cost function (described below). A wide range of social time preference (from 0 to 0.02) modulates the amount of weight given to the welfare of future generations relative to that of the current generation.

We explore the plausible range of mitigation cost by modulating the initial backstop cost of carbon such that, for a 2005 initialization, the ensemble range of mitigation cost for a given level of abatement in 2050 (evaluated as a fraction of Gross World Product, or GWP) is consistent with the range of mitigation costs observed in the IPCC AR5 emissions database^52^ (see Fig. 1).

### Baseline calculation

The baseline simulations are largely implemented as in^20^ - with all exogenous inputs identical to the baseline simulation in that paper. Additional data are used to initialize the model to 1970 conditions. Exogenous global population from^53^. Initial GDP data is taken from the World Bank^54^, global mean surface temperature from^55^ and global fossil fuel and land use emissions from^52^. Key variables from the resulting baseline simulation with default parameters are plotted in Supplemental Fig. A.1. Each parameter configuration exhibits a different global mean temperature evolution, but the baseline simulations have no climate damages nor abatement costs.

### Constrained simulations

Temperature constraints for the 1.5 and 2C scenarios are implemented by a semi-linear temperature damage function, such that the cost of exceeding the climate target increases from 0 to 100% of GWP above the desired target:

1 $${f}_{const}=\{\begin{array}{ll}\mathrm{0,} & {\rm{if}}\,T < {T}_{t}\\ \mathrm{(1/}r)(T-{T}_{t}), & {\rm{if}}\,{T}_{t}\le T < {T}_{t}+r\\ \mathrm{1,} & {\rm{if}}\,T\ge {T}_{t}+r\end{array}$$

where *T* is the global mean temperature above pre-industrial levels, *T*_*t*_ is the desired temperature target (1.5 or 2 degrees C in this case), and *r* is the ramping range in Celsius (where *r* = 0.5*C* is used in this case). The damage functions are illustrated in the Supplementary Material, Fig. A.2. The default DICE damage function is not active for these simulations.

The damage estimate is therefore not useful in itself, but forces the model to produce an optimal pathway (if possible) that does not exceed the desired temperature target and does not account for climate damages. Using a ramp function rather than a step function was found (empirically) to create stability for the solver by allowing the model to solve with short overshoots of the temperature target, however, with the chosen damage function the damage costs associated with the least cost solution are in practise negligible with respect to the abatement costs.

### Optimal solutions with climate damages

In the second set of experiments, the semi-linear climate damage function is replaced with the quadratic damage function used in the default model configuration. The quadratic coefficient is allowed to vary in the ensemble sufficiently to span the range of global damage estimates from a number of sources^5,10–15^ - allowing for damages to be significantly greater than the current range used in the IAMs which contributed to the 5th Assessment Report of the IPCC.

The model is then solved for the optimal growth and emissions pathway for each parameter configuration, with no exogenous constraint on temperature change.

## Supplementary information

Supplementary information
