# Supplementary material for: Assessing the costs of historical inaction on climate change
Source: Sci Rep. 2020 Jun 8;10:9173. doi: 10.1038/s41598-020-66275-4 (PMC7280186; doi:10.1038/s41598-020-66275-4)
Supplement: Supplementary file 1 — Supplementary information [file 41598_2020_66275_MOESM1_ESM.pdf]

Assessing the costs of historical inaction on climate change: Supplementary Material

Benjamin M. Sanderson and Brian C. O'Neill

Appendix A. Additional Tables and Figures

Model	Scenario	2050 Mitigation Costs (fraction GWP)	2050 Emissions (GtCO2)
GCAM 3.0	ROSE 450 LO Fos	0.01791	7.2957
GCAM 3.0	ROSE 550 HI Pop	0.018703	10.3947
IMAGE 2.4	AMPERE2-450-LowEI-HST	0.0067767	11.2514
IMAGE 2.4	AMPERE2-450-LowEI-LST	0.0071617	10.3572
IMAGE 2.4	AMPERE2-450-NucOff-OPT	0.012744	11.3918
IMAGE 2.4	EMF27-450-FullTech	0.012618	10.6135
IMAGE 2.4	EMF27-450-LimSW	0.012821	11.0729
IMAGE 2.4	EMF27-450-LowEI	0.0069482	11.3893
IMAGE 2.4	EMF27-450-NucOff	0.014689	10.611
IMAGE 2.4	LIMITS-450	0.011725	11.6182
IMAGE 2.4	LIMITS-RefPol-450	0.009976	11.9719
IMAGE 2.4	LIMITS-RefPol-450-EE	0.0088107	11.9719
IMAGE 2.4	LIMITS-RefPol-450-PC	0.009976	11.9719
IMAGE 2.4	LIMITS-StrPol-450	0.010097	11.9685
IMAGE 2.4 EMF22	EMF22 2.6 OS BECCS	0.014185	9.1169
MESSAGE V.1	EMF22 3.7 NTE	0.012887	11.45

Table A.1: Model Scenarios in the IPCC AR5 IAM database which support equivalently low abatement costs to those required in Figure 6 in order to reach the 2C target

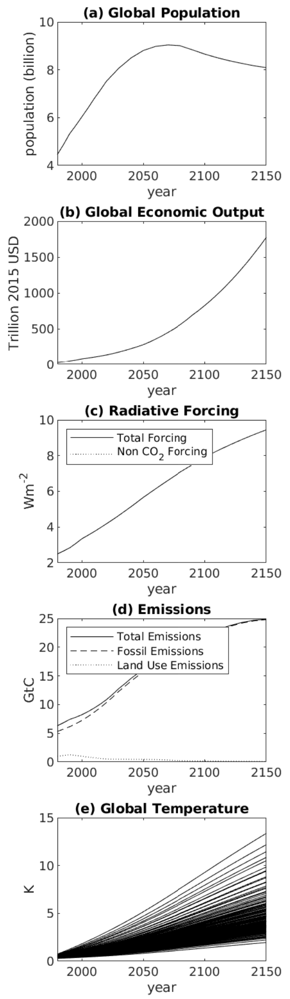

Figure A.1: Illustration of the baseline scenario used in the DICE model to inform this analysis for the default DICE parameters. The scenario corresponds to that used in the 2010 US social cost of carbon calculation [1]. Panel (a) is a model input assumption, panels (b)-(d) are model outcomes for the optimal solution without considering climate impacts on the economy or emissions mitigation. Panel (e) shows the range of temperature evolution given the prior uncertainty in Climate Sensitivity - but the baseline calculations have no climate damage costs.

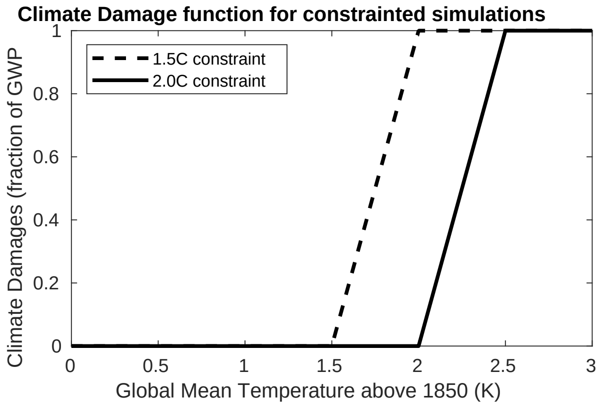

Figure A.2: Plot showing the climate damage function used to force the DICE model to solve for solutions which meet the 1.5 or 2 degree Paris temperature targets. Horizontal axis shows warming above pre-industrial levels, while the vertical axis shows climate damage function as a fraction of Gross World product.

References

[1] US Department of Energy. Final rule technical support document (tsd): energy efficiency program for commercial and industrial equipment: small electric motors, appendix 15a (by the interagency working group on social cost of carbon): "social cost of carbon for regulatory impact analysis under executive order 12866" (2010).
